# Ultrahigh‑resolution quantum dot light‑emitting diodes

**DOI:** 10.1093/nsr/nwag549

**Published:** 2026-08-29

**Authors:** Mengyuan Li, Xinyu Du, Wenxiang Xue, Jiahao Liu, Jinyi Lin, Wei Huang

**Affiliations:** State Key Laboratory of Flexible Electronics (LoFE) and Institute of Advanced Materials (IAM), Nanjing Tech University, China; State Key Laboratory of Flexible Electronics (LoFE) and Institute of Advanced Materials (IAM), Nanjing Tech University, China; State Key Laboratory of Flexible Electronics (LoFE) and Institute of Advanced Materials (IAM), Nanjing Tech University, China; State Key Laboratory of Flexible Electronics (LoFE) and Institute of Advanced Materials (IAM), Nanjing Tech University, China; State Key Laboratory of Flexible Electronics (LoFE) and Institute of Advanced Materials (IAM), Nanjing Tech University, China; State Key Laboratory of Flexible Electronics (LoFE) and Institute of Advanced Materials (IAM), Nanjing Tech University, China

## Abstract

This Perspective examines advanced patterning techniques for ultrahigh-resolution quantum dot light-emitting diodes, highlighting recent breakthroughs and the critical balance between resolution and device performance for next-generation near-eye displays.

Near-eye displays require retinal-level resolution (over 3000 PPI) to eliminate the screen-door effect and provide an immersive visual experience. However, scaling quantum dot light-emitting diodes (QLEDs) for ultrahigh-resolution microdisplays introduces several interconnected challenges: performance degradation during patterning process, inter-pixel gap regions leakage current and electric field concentration effects in submicron pixels [1], performance imbalances among red, green and blue (RGB) emitters [2], the submicron overlay precision required for full-color integration and the compatibility of QLEDs with complementary metal-oxide-semiconductor (CMOS) backplanes [3]. In addition, the fundamental differences in chemical stability between perovskite quantum dots (PeQDs) and traditional II–VI and III–V quantum dots (QDs) also result in differences in the applicability of various patterning strategies between these two classes of materials. Against this backdrop, recent breakthroughs have addressed these challenges through various mechanistic strategies, significantly advancing the development of ultrahigh-resolution QLEDs.

The electric field concentration effect at pixel edges is a key bottleneck facing patterned QLEDs at the submicron scale. Lin *et al*. developed a dual-action force dynamics strategy [4]. This method used nanoimprinting to form a honeycomb-like insulating skeleton (Fig. 1a) that blocks leakage paths, and regulated the dielectric constant of the insulating layer through TiO_2_ doping to match the QD layer, thereby eliminating electric field distortions within the microcavities. Based on this strategy, the device achieved a red external quantum efficiency (EQE) of 26.1% at 12 700 PPI, with an extrapolated T_95_ lifetime of 65 190 h at 1000 cd m^–2^, and CMOS compatibility has been verified on a 5600 PPI active-matrix display. Table S1 summarizes the key performance metrics of the patterning strategies. However, this process involves multiple layers of spin coating and precision transfer, has a narrow process window, and still poses challenges in controlling uniformity over large areas, which places high demands on yield control during mass production.

**Figure 1.**
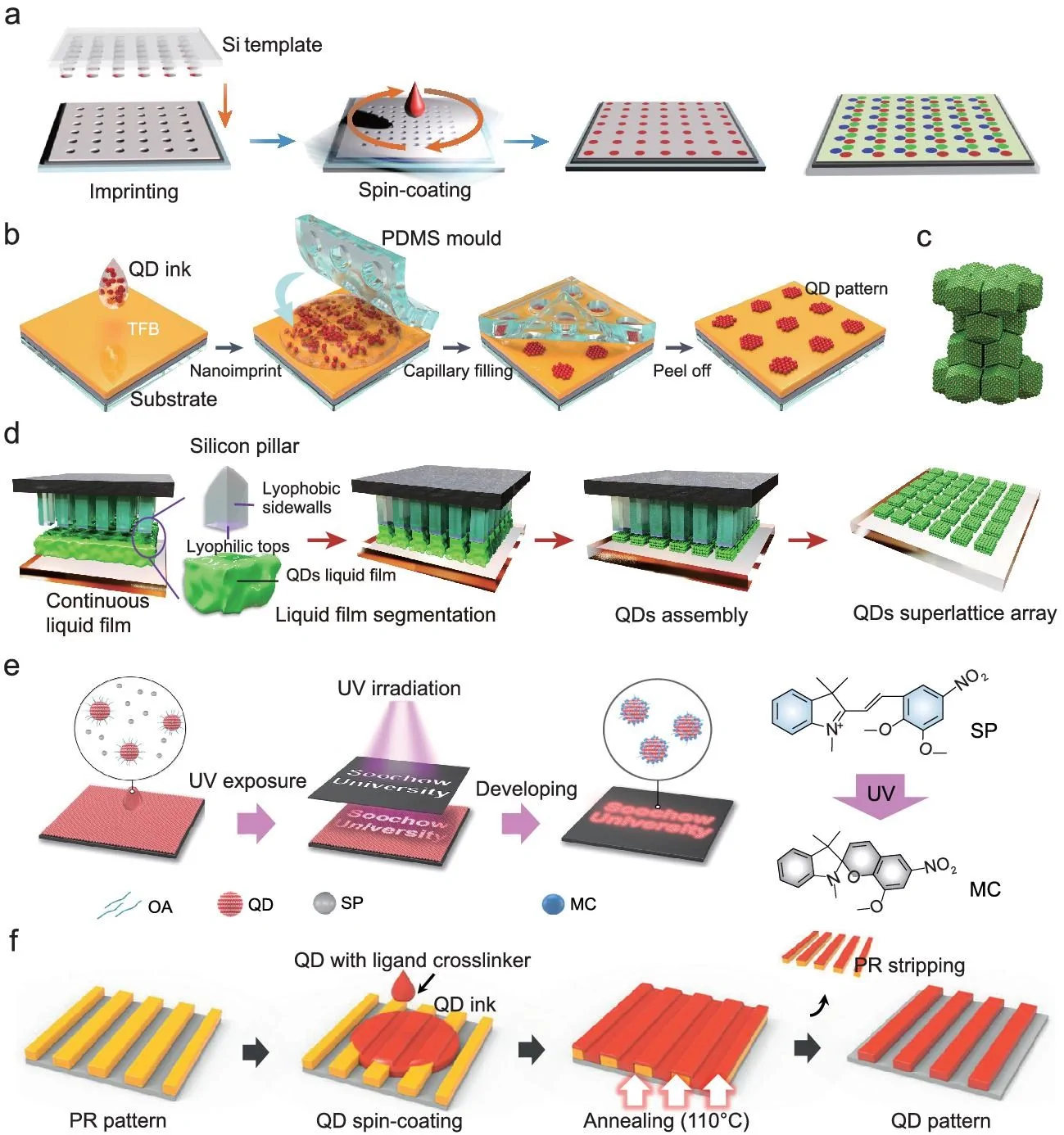
Overview of patterning strategies and key characterizations. (a) Schematic illustration of the nanoimprint-inverted transfer-printing process. Reproduced with permission from Ref [4]. Copyright: 2026 Springer Nature. (b) Schematic representation of the nanoimprint-based strategy via capillary interaction for the self-assembly of QD patterns. High-resolution QD arrays were obtained through direct nanoimprinting on poly (9,9-dioctylfluorene-co-N-(4-butylphenyl) diphenylamine) (TFB) followed by capillary-force-driven self-assembly. Reproduced with permission from Ref [5]. Copyright: 2026 Springer Nature. (c) Predicted hexagonal close packing (hcp) stacking formed by rhombic dodecahedral PeQDs. (d) Schematic of capillary liquid-bridge confined assembly for patterning PeQD superlattice pixels. (c, d) Reproduced with permission from Ref [6]. Copyright: 2026 Springer Nature. (e) Illustration of the QD patterning process with the successive steps of QD film preparation, UV exposure and developing processes. Reproduced with permission from Ref [7]. Copyright: 2026 Springer Nature. (f) Schematic illustrations of the photoresist-guided indirect (PIN) photopatterning process using low-temperature activatable ligand crosslinker. Reproduced with permission from Ref [8]. Copyright: 2026 Springer Nature.

The capillary action-induced self-assembly strategy proposed by Cao *et al*. aimed to explore the upper limit of patterning resolution [5]. This method utilized nanohole-array molds and capillary forces to achieve dense single-layer self-assembly of QDs by controlling the solvent evaporation rate (Fig. 1b). The single-layer structure effectively suppressed leakage current as pixel size decreases, providing a structural foundation for maintaining device performance. The resulting resolution of 169 333 PPI was the highest reported to date, with pixel sizes smaller than 100 nm, corresponding to an effective electroluminescent area of ∼4 mm^2^, and was compatible with flexible substrates. However, it is worth noting that this resolution exceeds the addressing capability of current active-matrix backplanes; currently, it can only be driven using a passive-matrix approach.

Both of the above strategies focus on the spatial arrangement of QDs, but the inherent structural disorder of the materials may limit performance improvements. The PeQD superlattice assembly reported by Zhang *et al*. addressed this issue by leveraging the material’s intrinsic structure (Fig. 1d) [6]. Through the synergistic stabilization of ligands and fluorine, CsPbBr_3_ rhombohedral dodecahedral QDs self-assemble into a hexagonal close packing superlattice with long-range order (Fig. 1c). The ordered structure effectively suppressed energy disorder, enabling band-like charge transport and enhancing electron coupling between QDs. Consequently, green perovskite QLEDs achieved a peak EQE (EQE_max_) of 30.9% and an extrapolated T_50_ exceeding 12 000 h at 100 cd m^–2^, representing a three-order-of-magnitude improvement over previously reported patterned PeQD devices, and have been integrated into a 352 × 430 active-matrix display. However, this strategy is currently applicable primarily to perovskite systems; its extension to traditional QDs still faces challenges related to the adaptation of nanocrystal shapes and surface chemistry.

The direct photoisomeric transformation patterning strategy proposed by Wu *et al*. redefined the logic of patterning at the chemical level, transforming photochemical reactions into a source of performance gains [7]. Under ultraviolet (UV) light, spiropyran isomerized into merocyanine, triggering ligand exchange on the QD surface (Fig. 1e). The resulting merocyanine simultaneously suppressed non-radiative energy transfer and passivated surface defects, resulting in a higher photoluminescence quantum yield for the patterned film compared to the original film. It achieved 15 800 PPI and 24.5% EQE (the highest record for directly photopatterned devices), and was compatible with both CdSe/ZnS and PeQDs, making it suitable for both rigid and flexible substrates. However, the resolution is limited by optical diffraction of UV light, with a minimum feature size of ∼0.8 μm.

Kim *et al*. proposed a photoresist-guided indirect patterning strategy that decouples pattern definition from QD cross-linking, thereby achieving a balance between pattern fidelity and QD performance [8]. This process first formed a patterned photoresist sacrificial layer, followed by the deposition of QDs and low-temperature

thermal cross-linking, and finally the stripping of the photoresist to reveal the QD pattern (Fig. 1f). The indirect process avoided direct damage to the QDs from UV light, achieving a line edge roughness of only 43 nm. The strategy enabled RGB patterns exceeding 4000 PPI and demonstrated the feasibility of multi-round overprinting through a 10 × 10 passive-matrix array. However, while this process involves multiple steps and is complex, it is compatible with existing semiconductor manufacturing equipment and is expected to improve mass production yield by leveraging established process expertise.

All of the above strategies followed the basic logic of arranging RGB subpixels side-by-side in the plane. However, the vertically stacked architecture offered a completely different design approach. The structures reported by Kim *et al*. [9] and Ko *et al*. [10], respectively, stack the RGB layers in the thickness direction, reducing the number of subpixels per pixel from three to two, which theoretically increases the aperture ratio by 50%. There are two specific implementation methods: one involves stacking a normal structure and an inverted structure within the same subpixel, emitting red or green light depending on the voltage polarity, and pairing it with a blue subpixel, and the other uses inkjet printing to pattern the bottom red and green layers, followed by a full-area coverage of the blue layer, thereby doubling the blue area. Although these two approaches avoid the overlay requirements of lateral fine patterning, they face challenges such as incompatibility between alternating-current drive and direct-current thin film transistor backplanes, as well as multi-layer interface engineering.

When assessing the applicability of these patterning strategies, differences in material systems are a factor that cannot be overlooked. Conventional QDs possess a protective core-shell structure with moderate ligand binding affinity, exhibiting a certain degree of tolerance to mild solvents, moderate heating and limited UV exposure. In contrast, PeQDs lack a protective shell, have weaker ligand binding and degrade rapidly in polar solvents. This fundamental difference means that patterning strategies validated in conventional QD systems often fail when directly applied to perovskite systems.

Looking beyond QLEDs patterning technology to the broader microdisplay field, QLEDs also face challenges from competing technologies such as organic light-emitting diodes (OLEDs), micro-LEDs and color-conversion architectures. For example, micro-LEDs face challenges such as efficiency loss and mass transfer yield issues during chip miniaturization; for OLEDs, efficiency roll-off at high brightness (especially for blue light) and the balance between long lifespan and high color purity remain key industry concerns; and color-conversion architectures face challenges related to light crosstalk and conversion efficiency. In contrast, QLEDs combine direct electroluminescence with solution-based patterning, offering the potential to circumvent some of these limitations. However, realizing this potential also depends on further breakthroughs in the efficiency and lifespan of blue QLEDs.

In summary, this Perspective systematically examines five advanced patterning techniques and two types of vertically stacked architectures for ultrahigh-resolution QLEDs. These works present distinct solutions addressing several key challenges.

These advances indicate that the fundamental trade-off between high resolution and high performance is gradually being resolved. However, with industrial application as the ultimate goal, several challenges still need to be addressed urgently. First, the efficiency and lifetime of blue QLEDs still fall short of those of red and green devices, which is currently the most prominent shortcoming in full-color displays. Second, achieving sub-100 nm overlay accuracy during sequential patterning steps, while avoiding damage to already deposited functional layers remains a major challenge for process integration. Third, the high-energy irradiation, polar solvents and high-temperature treatments involved in the patterning process can all cause irreversible damage to QDs, creating an urgent need to develop process solutions that physically or chemically isolate the QDs from these damage sources. Fourth, heavy-metal-free material systems (such as InP, ZnSeTe) require the development of dedicated, gentle patterning methods to meet increasingly stringent environmental requirements. Fifth, the compatibility of patterning processes with existing semiconductor production lines needs systematic evaluation, as this directly determines the feasibility of scaling these technologies from the laboratory to the factory. In the coming years, as these issues are gradually resolved, QLEDs are expected to emerge as a competitive technical solution in the near-eye display field, providing a viable path toward achieving retinal-level resolution and immersive visual experiences.

## Supplementary Material

nwag549_Supplemental_File
