# Supplementary material for: Ultrahigh‑resolution quantum dot light‑emitting diodes
Source: Natl Sci Rev. 2026 Aug 29;13(20):nwag549. doi: 10.1093/nsr/nwag549 (PMC13750179; doi:10.1093/nsr/nwag549)
Supplement: Supplementary file 1 [file nwag549_supplemental_file.pdf]

Supporting Information

PERSPECTIVE

CHEMISTRY

Ultrahigh-resolution quantum dot light-emitting diodes

Mengyuan Li, Xinyu Du, Wenxiang Xue, Jiahao Liu, Jinyi Lin* and Wei Huang*

State Key Laboratory of Flexible Electronics (LoFE) and Institute of Advanced Materials (IAM), Nanjing Tech University (NanjingTech), Nanjing 210009, China

***Corresponding authors.** iamjylin@njtech.edu.cn; vc@nwpu.edu.cn

Table S1. Comparison of key performance metrics of five patterning strategies for ultrahigh-resolution quantum dot light-emitting diodes (QLEDs).

	PPI_{max}	EQE_{max} (Red)	Operational lifetime	CMOS compatibility	Overlay precision	Scalability	Manufacturing complexity
**Lin et al** [1].	25400	26.1%	65,190 h (T_{95})	Yes	Submicron	Medium	High
**Cao et al** [2].	169333	17%	~7,970 h (T_{95})	No	Nanoscale	High	Medium
**Zhang et al** [3].	5080	-	>12,000 h (T_{50})	Yes	Micron	Medium	Medium
**Wu et al** [4].	15800	24.5%	2,214 h (T_{95})	No	Micron	High	Low
**Kim et al** [5].	>4000	~22%	-	No	Submicron	High	High

The lifetimes correspond to an initial luminance of 1,000 cd/m^2 for all studies except Zhang et al., where the initial luminance is 100 cd/m^2. The lifetime definition standards (T_{50} vs. T_{95}) and test conditions vary across studies, and direct comparisons should be made with caution.

It can be seen that a higher PPI does not equate to practical application value. Although Cao *et al* [2]. achieved a high PPI of 169,333, this resolution exceeded the addressing limits of current active-matrix backplanes, necessitating the use of passive-matrix drive. As a result, individual pixels cannot be controlled independently, and the effective display area was fundamentally constrained. In contrast, the work by Lin *et al* [1]. and Zhang *et al* [3]., although achieving PPI of 25,400 and 5,080 PPI, respectively, has made substantial progress at the system integration level, successfully demonstrating compatibility with complementary metal-oxide-semiconductor (CMOS) or thin film transistor driven backplanes. The comparison illustrates that the key to the practical application of ultrahigh-resolution patterning technology lies in the compatibility of pixel size with existing drive architectures.

1. Lin L, Wang J, Hu H *et al*. *Nature* 2026; **652**: 349–358.

2. Cao W, Tian S, Zhong C *et al*. *Nat Photon* 2026; **20**: 301–309.

3. Zhang C, Zeng Q, Li, H *et al*. *Nature* 2026; **652**: 1173–1180.

4. Wu C, Luo C, Huo Y *et al*. *Light Sci Appl* 2026; **15**: 157.

5. Kim H, Ham H, Lim C H *et al*. *Nat Commun* 2026; **17**: 4162.
